# HIV-1 *trans*-Infection Mediated by DCs: The Tip of the Iceberg of Cell-to-Cell Viral Transmission

**DOI:** 10.3390/pathogens11010039

**Published:** 2021-12-31

**Authors:** Daniel Perez-Zsolt, Dàlia Raïch-Regué, Jordana Muñoz-Basagoiti, Carmen Aguilar-Gurrieri, Bonaventura Clotet, Julià Blanco, Nuria Izquierdo-Useros

**Affiliations:** 1 IrsiCaixa AIDS Research Institute, Can Ruti Campus, 08916 Badalona, Spain; dperez@irsicaixa.es (D.P.-Z.); draich@irsicaixa.es (D.R.-R.); jmunoz@irsicaixa.es (J.M.-B.); caguilar@irsicaixa.es (C.A.-G.); bclotet@irsicaixa.es (B.C.); jblanco@irsicaixa.es (J.B.); 2 Infectious Diseases and Immunity Department, Faculty of Medicine, University of Vic-Central University of Catalonia (UVic-UCC), 08500 Vic, Spain; 3 Germans Trias i Pujol Research Institute (IGTP), Can Ruti Campus, 08916 Badalona, Spain

**Keywords:** DC, *trans*-infection, HIV-1, Ebola virus, SARS-CoV-2

## Abstract

HIV-1 cell-to-cell transmission is key for an effective viral replication that evades immunity. This highly infectious mechanism is orchestrated by different cellular targets that utilize a wide variety of processes to efficiently transfer HIV-1 particles. Dendritic cells (DCs) are the most potent antigen presenting cells that initiate antiviral immune responses, but are also the cells with highest capacity to transfer HIV-1. This mechanism, known as *trans*-infection, relies on the capacity of DCs to capture HIV-1 particles via lectin receptors such as the sialic acid-binding I-type lectin Siglec-1/CD169. The discovery of the molecular interaction of Siglec-1 with sialylated lipids exposed on HIV-1 membranes has enlightened how this receptor can bind to several enveloped viruses. The outcome of these interactions can either mount effective immune responses, boost the productive infection of DCs and favour innate sensing, or fuel viral transmission via *trans*-infection. Here we review these scenarios focusing on HIV-1 and other enveloped viruses such as Ebola virus or SARS-CoV-2.

## 1. Walking on Thin Ice: Cellular Mechanisms of HIV-1 Spread

Several viruses have the ability to hijack pre-existing mechanisms of cellular communication to facilitate direct cell-to-cell viral spread [1,2,3], and the human immunodeficiency virus type 1 (HIV-1) is not an exception [1,4]. Before the definition of the precise mechanisms of cell-to-cell viral transmission, early studies highlighted the increased efficiency of HIV-1 spread by cellular contacts as compared to the diffusion-limited movement of free viral particles, suggesting that cell-to-cell dissemination might be up to 1000 times more efficient [5]. However, the first detailed description of a stable cellular junction between infected and non-infected cells to facilitate viral spread, known as virological synapse (VS), was reported for the human T cell leukaemia virus type 1 (HTLV-1), which is inefficient at infecting T cells and requires cellular contacts for effective spread [6]. Soon after this description, several studies showed co-clustering of HIV-1 proteins with their receptors CD4 and CXCR4, together with a massive viral transmission at the stable interface formed between HIV-1-infected and non-infected CD4^+^ T cells [7,8], thus expanding the concept of vs. to HIV-1.

The relevance of this mechanism prompted extensive research in HIV-1 transmission allowing for the identification of several types of synapses involving: susceptible cells to infection, such as CD4^+^ T cells or macrophages [4,9]; non-susceptible cells to infection, such as endothelial cells or dendritic cells (DCs) [10,11]; and different mechanisms of membrane dynamics [12]. Aside from entering susceptible cells through envelope glycoprotein-mediated fusion at the plasma membrane, HIV-1 also accumulates into target cells using the intracellular or endocytic route that may involve active infection or passive transport of viruses. Several studies have shown that HIV-1 particles can be captured into intracellular compartments in different cell types, being particularly relevant in mature DCs [13]. High levels of viral endocytosis have also been observed during synaptic transmission of HIV-1 in both CD4^+^ T cells [8] and DCs after being in contact with HIV-1 infected cells [14].

In addition to these mechanisms of viral entry, other transport phenomena have been observed in HIV spread through cell-to-cell contacts. Trogocytosis (from the ancient Greek *trogo*, meaning “gnaw” or “nibble”) was described as the transfer of plasma membrane fragments from a presenting cell to a lymphocyte [15] and has been documented in T, B and natural killer cells both in vitro and in vivo [16]. Trogocytosis can be distinguished from other mechanisms of intercellular material exchange because it is a process that allows the rapid transference of intact cell-surface proteins accumulated at the synapse and can also allow for transmission of HIV-1 receptors [17] or for massive transfer of large amounts of Gag involving entire synaptic buttons [18]. Another mechanism that may be implicated in cell-to-cell HIV-1 transfer are nanotubes, long cytoplasmatic bridges that facilitate communication between cells [19,20]. Another mode of HIV-1 transfer involves the establishment of filopodial bridges between infected and target cells where viruses move along the outer surface of the bridge toward the target cell [21]. Both filopodia and nanotubes might allow transfer to distant cells, as observed not only for retroviruses, but also for multiple viral species, such as herpesvirus, papillomavirus, and vaccinia virus [22].

Among cellular contacts involved in HIV-1 spread, the T cell-T cell synapse has been deeply analysed and studied. In T cell-T cell synapses, the central supramolecular activation cluster (cSMAC) is formed by the binding of cell surface-expressed envelope glycoprotein to its receptor CD4 on the uninfected cell [23,24,25]. Here, cellular contacts between infected and non-infected CD4^+^ T cells recruit the CD4 receptor and coreceptors CXCR4 and CCR5 to the site of cell-to-cell contact in an actin-dependent manner [7], whereas envelope glycoprotein and Gag are recruited to the interface by a microtubule-dependent mechanism [26], finally leading to viral budding towards the contact area where the vs. is assembled. The direct passage of virus across vs. may be particularly relevant in lymphoid tissues, where cells are in close contact, as well as in epithelial or endothelial surfaces, where cellular synapses may favour initial steps of viral invasion [10]. Furthermore, viral traffic across vs. may have additional implications, as it may confer shielding to the virus physically and over time against the inhibitory action of antibodies or antivirals [27,28,29].

In addition to the VS, there is another type of synapse formed between antigen presenting cells (APCs) such as DCs and CD4^+^ T cells, which can even operate in the absence of productive infection of the donor APC. During antigen presentation, the formation of cognate DC:T cell conjugates or ‘immunological synapses’ is necessary for the activation of T cells [30,31]. Once activated, T cells proliferate and differentiate into effector cells, which mediate adaptive immune responses aimed to eliminate invading viruses [32]. Intriguingly, upon HIV-1 infection, the intimate cell-to-cell contacts formed between DCs and CD4^+^ T cells can boost viral transmission via the formation of an ‘infectious synapse’ [33] that allows for systemic HIV-1 dissemination. In this review we focus on how DCs, which are the most potent APCs found in our organism [34,35], are also the ones with greater capacity to boost HIV-1 transmission via a cell-to-cell transfer mechanism co-opted by other enveloped viruses.

## 2. Breaking the Ice: DCs Orchestrate Immune Responses against HIV-1 and Other Viruses

DCs act as pivotal players in the initiation of immunity against invading viruses [36,37], participating in both innate and adaptive immune responses. These cellular sentinels patrol distinct mucosae and, upon infection, viral sensing triggers rapid innate immune responses to contain viral spread. DC activation also elicits cellular migration towards secondary lymphoid tissues, where DCs acquire a fully mature phenotype and become competent for presenting antigens to T cells and activate them [34,35].

DCs form an integral part of innate immunity, along with other leukocytes and tissue-resident cells. DCs are present at the sites of pathogen invasion such as mucosal surfaces and the skin, and are among the first cells encountering these pathogens. DCs detect molecular patterns shared by broad groups of pathogens, termed pathogen-associated molecular patterns (PAMPs), which include viral RNA or DNA genomes, bacterial lipopolysaccharide (LPS) and yeast mannans [38,39]. DCs recognize these conserved motifs through pattern-recognition receptors (PRRs) [40]. A well-studied family of PRRs are Toll-like receptors (TLRs), which recognize a variety of ligands [41,42], each TLR having a particular sub-cellular localization and ligand specificity [43]. For instance, endosomal TLR7 and TLR8 recognize single-stranded RNA, while TLR9 binds DNA, and TLR4 recognizes LPS, an integral component of the outer membrane of gram-negative bacteria. Another group of PRRs found on DCs are C-type lectin receptors (CLRs), which include DC-SIGN (CD209), L-SIGN (CD299, Clec4M) and LSECtin (Clec4G), and recognize high mannose-containing glycans [44,45]. Within the group of I-type lectin receptors, the sialic acid-binding Ig-like lectins (Siglecs) are the best characterized members [46,47]. They are expressed by DCs, macrophages and monocytes and recognize sialic acids found on pathogens and also in host cells [48].

Viral recognition by DCs triggers the expression of genes involved in the secretion of cytokines and chemokines [49,50], which create a proinflammatory environment to eliminate or limit its replication. The main antiviral cytokines are type I interferons (IFNs), such as IFNα and IFNβ, and plasmacytoid DCs are major producers of these cytokines [51]. DCs that patrol mucosal surfaces display an immature status and can trap viruses at the entry sites, degrade them in endosomal lytic compartments and load pathogen-derived peptides onto molecules of the major histocompatibility complex (MHC). When this occurs, DCs become activated and migrate to the secondary lymphoid tissues [52], where DCs present viral-derived antigens to naïve T lymphocytes.

There are different ways of antigen presentation by DCs to T cells, depending partially on the origin of such antigens. Endogenous antigens are those expressed by the DC itself (for example viral proteins synthesized in the cytoplasm upon viral infection), and after proteasomal cleavage, the derived peptides are loaded onto MHC class I molecules and presented to CD8^+^ T cells [53]. In contrast, exogenous antigens are internalized by DCs through pinocytosis, phagocytosis and receptor-mediated endocytosis, processed by endosomal proteases, and the derived peptides are incorporated onto MHC class II molecules that also reach the cell surface [54]. MHC-II:peptide complexes are recognized by CD4^+^ T cells, which differentiate into several effector cell subtypes. In the context of viral infection these cells are mainly Th1 and T follicular helper cells [55], which prompt specific antiviral responses.

Of note, DCs have the unique capacity of presenting exogenous antigens to CD8^+^ T cells via MHC-I, a process known as ‘cross-presentation’ [56]. This mechanism allows antigen presentation to CD8^+^ T cells without productive DC infection, and is an efficient presentation pathway for viruses such as influenza A virus (IAV) [57,58] and HIV-1 [59]. Another non-classical antigen presentation pathway is that followed by endogenous peptides from measles virus [60], IAV [61] and HIV-1 [62], which are loaded onto MHC-II molecules instead of MHC-I molecules, being therefore presented to CD4^+^ T cells.

Despite the fined-tuned machinery for antigen presentation displayed by DCs, DC:T cell conjugates also represent a unique niche for viral transmission through the formation of infectious synapses [33], a mechanism extensively studied for HIV-1, that is also hijacked by other enveloped viruses.

## 3. When Immunity Is Put on Ice: DCs as Promoters of HIV-1 Cell-to-cell Transmission

**Figure 1:**
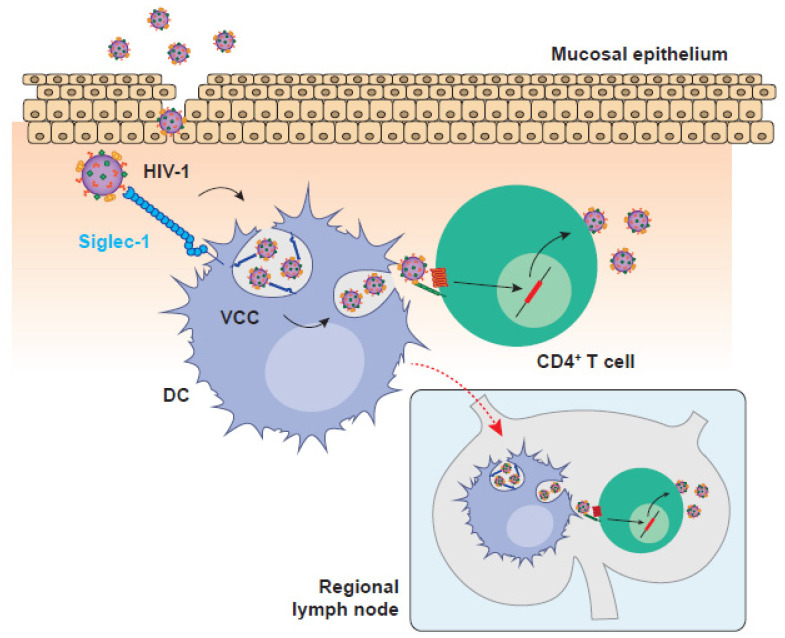
HIV-1 invasion is boosted by DC-mediated viral transmission in the mucosa and the migration to secondary lymphoid tissues. HIV-1 replication in the mucosa is facilitated by Siglec-1-expressing DCs that can mediate viral transmission to mucosal CD4^+^ T cells or migrate to secondary lymphoid tissues where the interaction with other target CD4^+^ T cells accelerates the settlement of systemic infection. HIV-1: human immunodeficiency virus type 1; VCC: viral containing compartment; DC: dendritic cell.

Although DCs orchestrate key innate and adaptive immune antiviral responses [36,37,63], HIV-1 and other viruses have evolved strategies to evade DC surveillance [64,65,66]. Indeed, viruses exploit DC function as a way to fuel infection of target cells, colonizing distant tissues as DCs migrate (Figure 1). Landmark studies carried out in the 90s in the laboratory of Ralph Steinman showed that the efficacy of HIV-1 infection of CD4^+^ T cells was increased when DCs were added in co-culture as compared to the transmission of cell-free viruses [67,68]. Noteworthy, DCs are non-permissive to HIV-1 infection, as they express low levels of viral receptor and co-receptors [69], efficiently degrade incoming viruses [70,71] and express several restriction factors such as SAMHD1 that interfere with viral replication [72,73,74,75,76,77]. However, these pioneering studies demonstrated that DCs can transmit a vigorous HIV-1 infection to bystander CD4^+^ T cells in the absence of productive viral replication on DCs, a mechanism of viral cell-to-cell transmission known as *trans*-infection [44,67].

*trans*-infection is one of the most potent viral transmission processes identified so far, but is only boosted when DC infection is restricted, as it is the case of HIV-1. *trans*-infection was initially attributed to the activity of a DC-specific intercellular adhesion molecule-3-grabbing non-integrin (DC-SIGN), a C-type lectin receptor expressed by DCs that recognizes the HIV-1 envelope glycoprotein [44,78]. However, several studies suggested that other receptors aside from DC-SIGN operated in HIV-1 transmission [79,80,81,82,83,84,85,86]. This was suspected because DC maturation greatly increased HIV-1 *trans*-infection capacity while it decreased the expression of DC-SIGN [86], and because antibodies directed against DC-SIGN were not able to consistently block HIV-1 transmission [82]. Such inconsistencies led to the identification, almost a decade ago, of the sialic acid-binding immunoglobulin-like lectin 1 (Siglec-1/CD169) as the key molecule for DC-mediated HIV-1 *trans*-infection [87,88].

Siglec-1, also termed sialoadhesin, is an I-type lectin expressed by APCs of myeloid origin such as DCs, macrophages and monocytes [87,88,89,90,91]. At a structural level, this receptor consists of different immunoglobulin-like domains or ‘sets’, all of them extracellular. The N-terminal V-set domain contains the ligand binding activity, while the 16 extracellular C2-set domains project the V-set domain out of the cell glycocalyx, allowing for the interaction with extracellular molecules [48,92,93]. Siglec-1 has affinity for sialic acid present in both N- and O-glycans, with a higher preference for α2-3 linkages [94]. These sugars are found in a variety of complex glycolipid molecules such as gangliosides GM1a and GM3, which are components of the cell and viral membranes. In particular, these gangliosides are present in the membrane of HIV-1, allowing for viral binding to DCs via Siglec-1 and the subsequent transmission to by-stander CD4^+^ T cells [87,88,95,96]. Siglec-1 avidity for sialylated ligands is increased upon clustering of thousands of gangliosides in the viral membrane [48].

HIV-1 *trans*-infection is a dynamic process that involves viral attachment to Siglec-1, internalization within a viral containing compartment (VCC), and viral release to the intercellular space during the formation of DC:CD4^+^ T cell infectious synapses [25,97]. Following Siglec-1 recognition, HIV-1 particles concentrate on the surface of DCs [14,98] and are internalized into non-classical and non-acidic endosomal VCC enriched in tetraspanins, MHC-II and Siglec-1 [89,97,99]. Of note, VCCs and their content remain connected to the extracellular milieu [14,98,99], which facilitates the transmission of trapped HIV-1 particles upon the formation of DC:CD4^+^ T cell contacts. Although the physiological function of VCCs remains unclear, it might be related to antigen dissemination and storage, as this compartment also serves as a depot of antigen-containing extracellular vesicles that are also captured by Siglec-1 and can prime adaptive immune responses [13,100,101]. Therefore, HIV-1 exploits a pre-exiting Siglec-1-dependent antigen dissemination pathway to gain access to target CD4^+^ T cells.

Aside from subverting antigen presentation, HIV-1 also exploits DC migratory capacity to spread systemically. This has led to the idea that DCs can operate as ‘Trojan Horses’ and disseminate HIV-1 from the portals of viral entry to lymphoid tissues [67,102]. HIV-1 is mainly acquired through sexual transmission [103] and early events of retroviral infection have been extensively studied in non-human primate models. Following early replication at the reproductive mucosa, DCs bearing retroviruses can be found in draining lymph nodes of different non-human primate models as soon as 24 h after vaginal challenge [104,105,106,107]. Noteworthy, viral spread does not only rely on the productive infection of DCs, but also on the transference of captured viral particles via *trans*-infection [108,109,110,111]. Indeed, through the ex vivo culture of cells derived from human cervical tissues, we demonstrated that this mechanism relies on Siglec-1 [112]. Of note, we identified the presence of Siglec-1-enriched VCCs in the biopsy of a viremic HIV-1^+^ patient [112], indicating that cervical DC-mediated HIV-1 *trans*-infection might be a relevant process for viral acquisition in vivo. Thus, *trans*-infection may be key to establishing HIV-1 infection in the mucosa, leading to systemic viral dissemination thanks to the migratory capacity of DCs. Yet, this mechanism originally described for HIV-1 is also used by many distinct viruses and can even lead to productive infection depending on the viral tropisms.

## 4. When DCs Go Ice-Cold Blooded: Infection by Other Viruses via Siglec-1

Sialylated gangliosides are incorporated during the budding process of different enveloped viruses [113,114,115,116], and can therefore interact with Siglec-1-expressing DCs and contribute to the pathogenesis of distinct viruses aside from HIV-1. The outcome of this early interaction may facilitate access to target cells via *trans*-infection, as we have already discussed for HIV-1 and will later comment for other viruses, but also mediate DC productive infection if viruses overcome resistance. Viral tropism is a key determinant to allow a productive *cis*-infection on DCs, or to limit susceptibility to favor dissemination via *trans*-infection, what can either contribute to boost antiviral immunity or favor viral immune evasion.

Paradoxically, in a murine model, Siglec-1 has a protective function capturing Friend virus complex (FVC), which reduces the pathogenesis by limiting viral spreading to erythroblasts and by triggering an effective cytotoxic T cell response via conventional DCs [117]. During murine herpesvirus-4 (MuHV-4) infection, readily infected subcapsular sinus (SCS) macrophages also protect target B lymphocytes by containing incoming viruses [117,118]. Similarly, vesicular stomatitis virus (VSV) infects Siglec-1 SCS macrophages to initiate a type I IFN response that prevents virus dissemination towards the central nervous system [119].

While the productive infection of HIV-1 on macrophages and a human pre-DC precursor is also facilitated by Siglec-1 [120,121], DCs are generally resistant to HIV-1 infection. Yet, resistance to HIV-1 infection can favor *trans*-infection and viral transmission in the absence of immune detection, as innate sensing and antigen presentation are delayed when DCs are not productively infected. Indeed, as opposed to HIV-1, HIV-2 productively infects DCs. This is because HIV-2 contains the viral accessory protein *vpx* that is able to counteract the activity of the cellular restriction factor SAMHD1 [72,73], which abrogates the retro-transcription of viral RNA into DNA and therefore precludes viral genome integration and infection. An intriguing feature of the natural course of HIV-2 infection is the better prognosis when compared to the evolution of HIV-1-infected individuals. This could be associated with its greater capacity to mount an anti-HIV-2 immune response, thanks to the productive infection of DCs. This triggers the presence of viral antigens that can be initially detected by innate sensors [122,123] and also effectively presented to CD4^+^ and CD8^+^ T cells.

Despite the triggering of immune signaling upon DC infection by viruses such as HIV-2, other highly pathogenic viruses also infect myeloid cells with a worst outcome. Ebola virus (EBOV) not only productively infects DCs, but also abrogates their function and prevents the initiation of adaptive immune responses, facilitating uncontrolled systemic virus replication [124]. Of note, Siglec-1 recognizes sialylated gangliosides on the surface of EBOV membranes and modulates the binding, uptake and trafficking of Ebola viruses towards VCCs, facilitating viral entry via the endosomal pathway [125]. This indeed has also been described for C-type lectins such as DC-SIGN, liver/lymph node sinusoidal endothelial C-type lectin (LSECtin) or proteins from the TIM/TAM family [126,127,128].

DCs are therefore susceptible to infection by some viruses, which counteract resistance to infection, as is the case of HIV-2, or have a myeloid cell tropism, as is the case of EBOV. Yet, DCs are also largely resistant to the infection of other viruses, as it happens with HIV-1. This resistance does however not hamper viral transmission, as *trans*-infection in the absence of productive infection is a highly infectious viral transmission pathway. Moreover, *trans*-infection subverts the immune surveillance of sentinel DCs expressing Siglec-1, which can be exploited by retroviruses and other enveloped viruses with restricted cellular tropism [117].

## 5. The Snowball Effect: Other Viruses including SARS-CoV-2 *Trans*-Infect via Siglec-1

As previously highlighted, the broad viral binding capacity of Siglec-1 relies on the recognition of sialylated gangliosides, which are also detected on the membranes of other retroviruses aside from HIV-1. This allows DC-mediated *trans*-infection of other viruses via Siglec-1. Indeed, the murine leukemia virus (MLV) relies on Siglec-1-mediated capture for *trans*-infection of permissive lymphocytes [129,130]. MLV virions are captured via Siglec-1 on sinus-lining macrophages that promote dissemination through synaptic contacts to permissive T cells and, more efficiently, into B cells [129,130]. Siglec-1 viral recognition is also extended to other families of enveloped viruses, including those from the *Paramyxoviridae* family such as Nipah and Hendra viruses. These viruses are also recognized by Siglec-1 leading to an enhanced viral capture on activated DCs that allows *trans*-infection of T cells [131].

**Figure 2:**
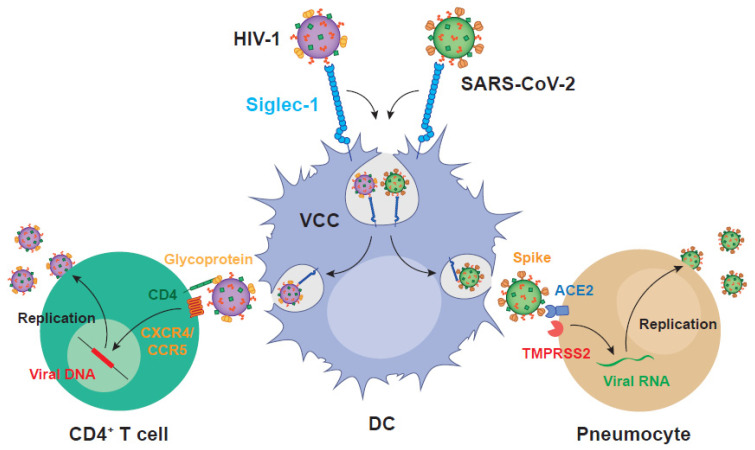
DCs mediate *trans*-infection of HIV-1 and SARS-CoV-2 to target cells via Siglec-1. Viral membrane ganglioside recognition of both HIV-1 and SARS-CoV-2 particles triggers VCC formation and effective transfer to susceptible target cells that become productively infected via viral glycoprotein interaction with CD4^+^ receptor and coreceptors in the case of HIV-1 and ACE2 and TMPRSS2 in the case of SARS-CoV-2. HIV-1: human immunodeficiency virus type 1; SARS-CoV-2: severe acute respiratory syndrome coronavirus 2; VCC: viral containing compartment; DC: dendritic cell; CXCR4: CXC chemokine receptor type 4; CCR5: CC chemokine receptor 5; ACE2: angiotensin-converting enzyme 2; TMPRSS2: transmembrane protease serine 2.

Gangliosides are also an integral part of the viral membranes of coronaviruses [132]. Thus, it is not surprising that SARS-CoV-2 is effectively *trans*-infected via Siglec-1 by DCs to ACE2-and TMPRSS2-expressingcells [132] in a mechanism of cell-to-cell transmission that parallels that previously described for HIV-1 (Figure 2). As Siglec-1-mediated viral transmission is not dependent on the recognition of the viral spike protein and relies on the interaction with viral membrane gangliosides, it is equally effective for different SARS-CoV-2 variants of concern [132]. Yet, in the case of SARS-CoV-2, further work should address if sialylated moieties associated to the abundantly expressed viral spike glycoprotein could be also implicated in Siglec-1 recognition. In human biopsies from COVID-19 patients, analysis of single cell data has shown that myeloid cells including DCs contain higher amounts of SARS-CoV-2 RNA, but lack ACE2, TMPRSS2, or classical SARS-CoV-2 entry factors [133]. These results clearly point out to the role of lectins such as Siglec-1 in trapping viruses in vivo, as already observed in non-human primate models [132].

Other respiratory enveloped viruses such as the human respiratory syncytial virus (RSV), the human metapneumovirus (HMPV) and other coronaviruses are also strong candidates to interact with Siglec-1 via ganglioside recognition. Indeed, this has been already described for the porcine reproductive and respiratory syndrome virus (PRRSV), which interacts with Siglec-1 on the surface of alveolar macrophages [134]. Yet, other respiratory viruses such as influenza lack gangliosides on their envelopes due to the activity of viral neuraminidases [135], and will therefore escape to Siglec-1 recognition.

It is important, however, to highlight that Siglec-1 viral recognition does not always lead to effective *trans*-infection, as both HIV-1 or SARS-CoV-2 are poorly transmitted by Siglec-1-expressing macrophages [89,132]. These APCs quickly degrade incoming viruses and are therefore less capable of transferring infectivity to bystander cells [89]. Thus, the outcome of Siglec-1 viral interaction depends on the cellular context and the activation status of the cells expressing this lectin. Dissecting the dual role of this lectin, which can favor viral dissemination but also contain infection while triggering antiviral immunity will be key to implement future treatments.

Despite the largely acknowledged relevance of cell-to-cell viral spread mechanisms in vivo, the exact contribution of Siglec-1-mediated *trans*-infection and other virus-mediated synapses is not yet defined. The ability of viral proteins to generate virological synapses and their cellular tropism are probably among the main factors that regulate both mechanisms. However, at least for HIV-1, current knowledge highlights relevant differences between them. vs. formed between infected and uninfected T cells are associated with cell death events involving apoptosis, autophagy or pyroptosis [136,137,138,139] but not necessarily with active viral replication [140]. In contrast, Siglec-1 *trans*-infection takes advantage of activation signals provided by DCs to CD4^+^ T cells, which promote active infection of target cells as suggested by the preferential infection of HIV-1-specific CD4^+^ T cells [141]. Further studies using relevant animal models will bring light into the relative contribution of Siglec-1-dependent and independent mechanisms of cell-to-cell transmission of HIV-1 and other viruses.

## 6. Melting the Ice: Concluding Remarks

Since the pioneering studies of the laboratory of Ralph Steinman deciphered the ability of DCs to *trans*-infect HIV-1 in the absence of productive infection [67], many reports have underscored the molecular pathways behind this mechanism, illustrating not only which lectin receptors are implicated in this viral transmission process, but also which restriction factors limit viral infectivity and contribute to *trans*-infection. Despite the molecular insights gained in recent years, which have allowed to demonstrate the role of *trans*-infection as a robust mechanism for viral dissemination within tissues in vivo, we still lack efficacious antiviral therapies to counteract this process. Recent neutralizing antibodies developed against SARS-CoV-2 interfere with this lectin-dependent pathway that enhances ACE2-dependent coronavirus infection [142]. Antibodies targeting Siglec-1 have also demonstrated efficacy at blocking MLV capture at the lymph node and spleen in mice [130]. In vitro, Siglec-1 blockage by antibodies has also disrupted HIV-1 and EBOV binding and uptake into activated DCs [87,88,125]. Given the growing number of enveloped viruses aside from HIV-1 that are able to subvert DCs for efficient dissemination, future work should implement strategies to block this pathway and contain the pathogenesis of known enveloped viruses and those to come.

## Data Availability

All data in this review is cited.

## References

[1] Bracq L., Xie M., Benichou S., Bouchet J. (2018). Mechanisms for Cell-to-Cell Transmission of HIV-1. Front. Immunol..

[2] Cifuentes-Munoz N., El Najjar F., Dutch R.E. (2020). Viral Cell-to-Cell Spread: Conventional and Non-Conventional Ways. Adv. Virus Res..

[3] Barretto N., Sainz B., Hussain S., Uprichard S.L. (2014). Determining the Involvement and Therapeutic Implications of Host Cellular Factors in Hepatitis C Virus Cell-to-Cell Spread. J. Virol..

[4] Puigdomènech I., Massanella M., Cabrera C., Clotet B., Blanco J. (2009). On the Steps of Cell-to-Cell HIV Transmission between CD4 T Cells. Retrovirology.

[5] Sato H., Orenstein J., Dimitrov D., Martin M. (1992). Cell-to-Cell Spread of HIV-1 Occurs within Minutes and May Not Involve the Participation of Virus Particles. Virology.

[6] Igakura T., Stinchcombe J.C., Goon P.K.C., Taylor G.P., Weber J.N., Griffiths G.M., Tanaka Y., Osame M., Bangham C.R.M. (2003). Spread of HTLV-I between Lymphocytes by Virus-Induced Polarization of the Cytoskeleton. Science.

[7] Jolly C., Kashefi K., Hollinshead M., Sattentau Q.J. (2004). HIV-1 Cell to Cell Transfer across an Env-Induced, Actin-Dependent Synapse. J. Exp. Med..

[8] Blanco J., Bosch B., Fernández-Figueras M.T., Barretina J., Clotet B., Esté J.A. (2004). High Level of Coreceptor-Independent HIV Transfer Induced by Contacts between Primary CD4 T Cells. J. Biol. Chem..

[9] Bracq L., Xie M., Lambelé M., Vu L.-T., Matz J., Schmitt A., Delon J., Zhou P., Randriamampita C., Bouchet J. (2017). T Cell-Macrophage Fusion Triggers Multinucleated Giant Cell Formation for HIV-1 Spreading. J. Virol..

[10] Alfsen A., Yu H., Magérus-Chatinet A., Schmitt A., Bomsel M. (2005). HIV-1-Infected Blood Mononuclear Cells Form an Integrin- and Agrin-Dependent Viral Synapse to Induce Efficient HIV-1 Transcytosis across Epithelial Cell Monolayer. Mol. Biol. Cell.

[11] Izquierdo-Useros N., Naranjo-Gómez M., Erkizia I., Puertas M.C., Borràs F.E., Blanco J., Martinez-Picado J. (2010). HIV and Mature Dendritic Cells: Trojan Exosomes Riding the Trojan Horse?. PLoS Pathog..

[12] Barroso-González J., García-Expósito L., Puigdomènech I., de Armas-Rillo L., Machado J.D., Blanco J., Valenzuela-Fernández A. (2011). Viral Infection: Moving through Complex and Dynamic Cell-Membrane Structures. Commun. Integr. Biol..

[13] Izquierdo-Useros N., Naranjo-Gómez M., Archer J., Hatch S.C., Erkizia I., Blanco J., Borràs F.E., Puertas M.C., Connor J.H., Fernádez-Figueras M.T. (2009). Capture and Transfer of HIV-1 Particles by Mature Dendritic Cells Converges with the Exosome-Dissemination Pathway. Blood.

[14] Izquierdo-Useros N., Esteban O., Rodriguez-Plata M.T., Erkizia I., Prado J.G., Blanco J., García-Parajo M.F., Martinez-Picado J. (2011). Dynamic Imaging of Cell-Free and Cell-Associated Viral Capture in Mature Dendritic Cells. Traffic.

[15] Joly E., Hudrisier D. (2003). What Is Trogocytosis and What Is Its Purpose?. Nat. Immunol..

[16] Hudrisier D., Bongrand P. (2002). Intercellular Transfer of Antigen-Presenting Cell Determinants onto T Cells: Molecular Mechanisms and Biological Significance. FASEB J..

[17] Hudrisier D., Aucher A., Puigdomnech I., Joly E., Clotet B., Blanco J., Puigdomènech I., Joly E., Clotet B., Hudrisier D. (2010). Could CD4 Capture by CD8+ T Cells Play a Role in Hiv Spreading?. J. Biomed. Biotechnol..

[18] Hübner W., McNerney G.P., Chen P., Dale B.M., Gordon R.E., Chuang F.Y.S., Li X.-D.D., Asmuth D.M., Huser T., Chen B.K. (2009). Quantitative 3D Video Microscopy of HIV Transfer across T Cell Virological Synapses. Science.

[19] Davis D.M., Sowinski S. (2008). Membrane Nanotubes: Dynamic Long-Distance Connections between Animal Cells. Nat. Rev. Mol. Cell Biol..

[20] Rudnicka D., Feldmann J., Porrot F., Wietgrefe S., Guadagnini S., Prévost M.-C.C., Estaquier J., Haase A.T., Sol-Foulon N., Schwartz O. (2009). Simultaneous Cell-to-Cell Transmission of Human Immunodeficiency Virus to Multiple Targets through Polysynapses. J. Virol..

[21] Sherer N.M., Lehmann M.J., Jimenez-Soto L.F., Horensavitz C., Pypaert M., Mothes W. (2007). Retroviruses Can Establish Filopodial Bridges for Efficient Cell-to-Cell Transmission. Nat. Cell Biol..

[22] Sherer N.M., Mothes W. (2008). Cytonemes and Tunneling Nanotubules in Cell-Cell Communication and Viral Pathogenesis. Trends Cell Biol..

[23] Piguet V., Sattentau Q. (2004). Dangerous Liaisons at the Virological Synapse. J. Clin. Investig..

[24] Puigdomènech I., Massanella M., Izquierdo-Useros N., Ruiz-Hernandez R., Curriu M., Bofill M., Martinez-Picado J., Juan M., Clotet B., Blanco J. (2008). HIV Transfer between CD4 T Cells Does Not Require LFA-1 Binding to ICAM-1 and Is Governed by the Interaction of HIV Envelope Glycoprotein with CD4. Retrovirology.

[25] Rodriguez-Plata M.T., Puigdomènech I., Izquierdo-Useros N., Puertas M.C., Carrillo J., Erkizia I., Clotet B., Blanco J., Martinez-Picado J. (2013). The Infectious Synapse Formed between Mature Dendritic Cells and CD4(+) T Cells Is Independent of the Presence of the HIV-1 Envelope Glycoprotein. Retrovirology.

[26] Jolly C., Mitar I., Sattentau Q.J. (2007). Requirement for an Intact T-Cell Actin and Tubulin Cytoskeleton for Efficient Assembly and Spread of Human Immunodeficiency Virus Type 1. J. Virol..

[27] Massanella M., Puigdoménech I., Cabrera C., Fernandez-Figueras M.T., Aucher A., Gaibelet G., Hudrisier D., García E., Bofill M., Clotet B. (2009). Antigp41 Antibodies Fail to Block Early Events of Virological Synapses but Inhibit HIV Spread between T Cells. Aids.

[28] Abela I.A., Berlinger L., Schanz M., Reynell L., Günthard H.F., Rusert P., Trkola A. (2012). Cell-Cell Transmission Enables HIV-1 to Evade Inhibition by Potent CD4bs Directed Antibodies. PLoS Pathog..

[29] Jolly C., Booth N.J., Neil S.J.D. (2010). Cell-Cell Spread of Human Immunodeficiency Virus Type 1 Overcomes Tetherin/BST-2-Mediated Restriction in T Cells. J. Virol..

[30] Dustin M.L., Chakraborty A.K., Shaw A.S. (2010). Understanding the Structure and Function of the Immunological Synapse. Cold Spring Harb. Perspect. Biol..

[31] Basu R., Huse M. (2017). Mechanical Communication at the Immunological Synapse. Trends Cell Biol..

[32] Hildreth A.D., O’sullivan T.E. (2019). Tissue-Resident Innate and Innate-like Lymphocyte Responses to Viral Infection. Viruses.

[33] Piguet V., Steinman R.M. (2007). The Interaction of HIV with Dendritic Cells: Outcomes and Pathways. Trends Immunol..

[34] Steinman R.M. (1991). The Dendritic Cell System and Its Role in Immunogenicity. Annu. Rev. Immunol..

[35] Banchereau J., Steinman R.M. (1998). Dendritic Cells and the Control of Immunology. Nature.

[36] Paludan C., Schmid D., Landthaler M., Vockerodt M., Kube D., Tuschl T., Münz C. (2005). Endogenous MHC Class II Processing of a Viral Nuclear Antigen after Autophagy. Science.

[37] Wilson N.S., Behrens G.M.N., Lundie R.J., Smith C.M., Waithman J., Young L., Forehan S.P., Mount A., Steptoe R.J., Shortman K.D. (2006). Systemic Activation of Dendritic Cells by Toll-like Receptor Ligands or Malaria Infection Impairs Cross-Presentation and Antiviral Immunity. Nat. Immunol..

[38] Janeway C.A., Medzhitov R. (2002). Innate Immune Recognition. Annu. Rev. Immunol..

[39] Cui J., Chen Y., Wang H.Y., Wang R.F. (2014). Mechanisms and Pathways of Innate Immune Activation and Regulation in Health and Cancer. Hum. Vaccines Immunother..

[40] Janeway C.A. (2013). Pillars Article: Approaching the Asymptote? Evolution and Revolution in Immunology. Cold Spring Harb Symp Quant Biol. 1989. 54: 1-13. J. Immunol..

[41] Medzhitov R., Preston-Hurlburt P., Janeway C.A. (1997). A Human Homologue of the Drosophila Toll Protein Signals Activation of Adaptive Immunity. Nature.

[42] Poltorak A., Smirnova I., He X., Liu M.-Y., Van Huffel C., Birdwell D., Alejos E., Silva M., Du X., Thompson P. (1998). Genetic and Physical Mapping of TheLpsLocus: Identification of the Toll-4 Receptor as a Candidate Gene in the Critical Region. Blood Cells Mol. Dis..

[43] O’Neill L.A.J., Golenbock D., Bowie A.G. (2013). The History of Toll-like Receptors—Redefining Innate Immunity. Nat. Rev. Immunol..

[44] Geijtenbeek T.B.H., Kwon D.S., Torensma R., van Vliet S.J., van Duijnhoven G.C.F., Middel J., Cornelissen I.L.M.H.A., Nottet H.S.L.M., KewalRamani V.N., Littman D.R. (2000). DC-SIGN, a Dendritic Cell-Specific HIV-1-Binding Protein That Enhances Trans-Infection of T Cells. Cell.

[45] Zhang F., Ren S., Zuo Y. (2014). DC-SIGN, DC-SIGNR and LSECtin: C-Type Lectins for Infection. Int. Rev. Immunol..

[46] Powell L.D., Varki A. (1995). I-Type Lectins. J. Biol. Chem..

[47] Angata T., Brinkman-Van der Linden E.C.M. (2002). I-Type Lectins. Biochim. Biophys. Acta (BBA)—Gen. Subj..

[48] Crocker P.R., Paulson J.C., Varki A. (2007). Siglecs and Their Roles in the Immune System. Nat. Rev. Immunol..

[49] Collin M., Bigley V. (2018). Human Dendritic Cell Subsets: An Update. Immunology.

[50] Rhodes J.W., Tong O., Harman A.N., Turville S.G. (2019). Human Dendritic Cell Subsets, Ontogeny, and Impact on HIV Infection. Front. Immunol..

[51] Siegal F.P., Kadowaki N., Shodell M., Fitzgerald-Bocarsly P.A., Shah K., Ho S., Antonenko S., Liu Y.-J. (1999). The Nature of the Principal Type 1 Interferon-Producing Cells in Human Blood. Science.

[52] Worbs T., Hammerschmidt S.I., Förster R. (2017). Dendritic Cell Migration in Health and Disease. Nat. Rev. Immunol..

[53] Hashimoto M., Im S.J., Araki K., Ahmed R. (2019). Cytokine-Mediated Regulation of CD8 T-Cell Responses during Acute and Chronic Viral Infection. Cold Spring Harb. Perspect. Biol..

[54] Mantegazza A.R., Magalhaes J.G., Amigorena S., Marks M.S. (2013). Presentation of Phagocytosed Antigens by MHC Class I and II. Traffic.

[55] Huang Q., Hu J., Tang J., Xu L., Ye L. (2019). Molecular Basis of the Differrentiation and Function of Virus Specific Follicular Helper CD4+ T Cells. Front. Immunol..

[56] Joffre O.P., Segura E., Savina A., Amigorena S. (2012). Cross-Presentation by Dendritic Cells. Nat. Rev. Immunol..

[57] Smed-Sörensen A., Chalouni C., Chatterjee B., Cohn L., Blattmann P., Nakamura N., Delamarre L., Mellman I. (2012). Influenza a Virus Infection of Human Primary Dendritic Cells Impairs Their Ability to Cross-Present Antigen to CD8 T Cells. PLoS Pathog..

[58] Bender A., Bui L.K., Feldman M.A., Larsson M., Bhardwaj N. (1995). Inactivated Influenza Virus, When Presented on Dendritic Cells, Elicits Human CD8+ Cytolytic T Cell Responses. J. Exp. Med..

[59] Larsson M., Fonteneau J.-F., Lirvall M., Haslett P., Lifson J.D., Bhardwaj N. (2002). Activation of HIV-1 Specific CD4 and CD8 T Cells by Human Dendritic Cells: Roles for Cross-Presentation and Non-Infectious HIV-1 Virus. AIDS.

[60] JACOBSON S., SEKALY R.P., BELLINI W.J., JOHNSON C.L., McFARLAND H.F., LONG E.O. (1988). Recognition of Intracellular Measles Virus Antigens by HLA Class II Restricted Measles Virus-Specific Cytotoxic T Lymphocytes. Ann. N. Y. Acad. Sci..

[61] Miller M.A., Ganesan A.P.V., Luckashenak N., Mendonca M., Eisenlohr L.C. (2015). Endogenous Antigen Processing Drives the Primary CD4+ T Cell Response to Influenza. Nat. Med..

[62] Coulon P.-G., Richetta C., Rouers A., Blanchet F.P., Urrutia A., Guerbois M., Piguet V., Theodorou I., Bet A., Schwartz O. (2016). HIV-Infected Dendritic Cells Present Endogenous MHC Class II–Restricted Antigens to HIV-Specific CD4+ T Cells. J. Immunol..

[63] Norbury C.C., Malide D., Gibbs J.S., Bennink J.R., Yewdell J.W. (2002). Visualizing Priming of Virus-Specific CD8+ T Cells by Infected Dendritic Cells in Vivo. Nat. Immunol..

[64] Pollara G., Kwan A., Newton P.J., Handley M.E., Chain B.M., Katz D.R. (2005). Dendritic Cells in Viral Pathogenesis: Protective or Defective?. Int. J. Exp. Pathol..

[65] Finlay B.B., McFadden G. (2006). Anti-Immunology: Evasion of the Host Immune System by Bacterial and Viral Pathogens. Cell.

[66] Rescigno M. (2015). Dendritic Cell Functions: Learning from Microbial Evasion Strategies. Semin. Immunol..

[67] Cameron P.U., Freudenthal P.S., Barker J.M., Gezelter S., Inaba K., Steinman R.M. (1992). Dendritic Cells Exposed to Human Immunodeficiency Virus Type-1 Transmit a Vigorous Cytopathic Infection to CD4+ T Cells. Science.

[68] Pope M., Betjes M.G.H., Romani N., Hirmand H., Cameron P.U., Hoffman L., Gezelter S., Schuler G., Steinman R.M. (1994). Conjugates of Dendritic Cells and Memory T Lymphocytes from Skin Facilitate Productive Infection with HIV-1. Cell.

[69] Lee B., Sharron M., Montaner L.J., Weissman D., Doms R.W. (1999). Quantification of CD4, CCR5, and CXCR4 Levels on Lymphocyte Subsets, Dendritic Cells, and Differentially Conditioned Monocyte-Derived Macrophages. Proc. Natl. Acad. Sci. USA.

[70] Turville S.G., Santos J.J., Frank I., Cameron P.U., Wilkinson J., Miranda-Saksena M., Dable J., Stössel H., Romani N., Piatak M. (2004). Immunodeficiency Virus Uptake, Turnover, and 2-Phase Transfer in Human Dendritic Cells. Blood.

[71] Moris A., Nobile C., Buseyne F., Porrot F., Abastado J.P., Schwartz O. (2004). DC-SIGN Promotes Exogenous MHC-I-Restricted HIV-1 Antigen Presentation. Blood.

[72] Laguette N., Sobhian B., Casartelli N., Ringeard M., Chable-Bessia C., Ségéral E., Yatim A., Emiliani S., Schwartz O., Benkirane M. (2011). SAMHD1 Is the Dendritic- and Myeloid-Cell-Specific HIV-1 Restriction Factor Counteracted by Vpx. Nature.

[73] Hrecka K., Hao C., Gierszewska M., Swanson S.K., Kesik-Brodacka M., Srivastava S., Florens L., Washburn M.P., Skowronski J. (2011). Vpx Relieves Inhibition of HIV-1 Infection of Macrophages Mediated by the SAMHD1 Protein. Nature.

[74] Lahouassa H., Daddacha W., Hofmann H., Ayinde D., Logue E.C., Dragin L., Bloch N., Maudet C., Bertrand M., Gramberg T. (2012). SAMHD1 Restricts the Replication of Human Immunodeficiency Virus Type 1 by Depleting the Intracellular Pool of Deoxynucleoside Triphosphates. Nat. Immunol..

[75] Sheehy A.M., Gaddis N.C., Choi J.D., Malim M.H. (2002). Isolation of a Human Gene That Inhibits HIV-1 Infection and Is Suppressed by the Viral Vif Protein. Nature.

[76] Mariani R., Chen D., Schröfelbauer B., Navarro F., König R., Bollman B., Münk C., Nymark-McMahon H., Landau N.R. (2003). Species-Specific Exclusion of APOBEC3G from HIV-1 Virions by Vif. Cell.

[77] Mangeat B., Turelli P., Caron G., Friedli M., Perrin L., Trono D. (2003). Broad Antiretroviral Defence by Human APOBEC3G through Lethal Editing of Nascent Reverse Transcripts. Nature.

[78] Geijtenbeek T.B.H., Tornsma R., van Vliet S.J., van Duijnhoven G.C.F., Adema G.J., van Kooyk Y., Figdor C.G. (2000). Identification of DC-SIGN, a Novel Dendritic Cell-Specific ICAM-3 Receptor That Supports Primary Immune Responses. Cell.

[79] Turville S.G., Cameron P.U., Handley A., Lin G., Pöhlmann S., Doms R.W., Cunningham A.L. (2002). Diversity of Receptors Binding HIV on Dendritic Cell Subsets. Nat. Immunol..

[80] Wu L., Bashirova A.A., Martin T.D., Villamide L., Mehlhop E., Chertov A.O., Unutmaz D., Pope M., Carrington M., KewalRamani V.N. (2002). Rhesus Macaque Dendritic Cells Efficiently Transmit Primate Lentiviruses Independently of DC-SIGN. Proc. Natl. Acad. Sci. USA.

[81] Gummuluru S., Rogel M., Stamatatos L., Emerman M. (2003). Binding of Human Immunodeficiency Virus Type 1 to Immature Dendritic Cells Can Occur Independently of DC-SIGN and Mannose Binding C-Type Lectin Receptors via a Cholesterol-Dependent Pathway. J. Virol..

[82] Trumpfheller C., Park C.G., Finke J., Steinman R.M., Granelli-Piperno A. (2003). Cell Type-Dependent Retention and Transmission of HIV-1 by DC-SIGN. Int. Immunol..

[83] Granelli-Piperno A., Pritsker A., Pack M., Shimeliovich I., Arrighi J.-F., Park C.G., Trumpfheller C., Piguet V., Moran T.M., Steinman R.M. (2005). Dendritic Cell-Specific Intercellular Adhesion Molecule 3-Grabbing Nonintegrin/CD209 Is Abundant on Macrophages in the Normal Human Lymph Node and Is Not Required for Dendritic Cell Stimulation of the Mixed Leukocyte Reaction. J. Immunol..

[84] Boggiano C., Manel N., Littman D.R. (2007). Dendritic Cell-Mediated Trans-Enhancement of Human Immunodeficiency Virus Type 1 Infectivity Is Independent of DC-SIGN. J. Virol..

[85] Wang J.-H., Janas A.M., Olson W.J., Wu L. (2007). Functionally Distinct Transmission of Human Immunodeficiency Virus Type 1 Mediated by Immature and Mature Dendritic Cells. J. Virol..

[86] Izquierdo-Useros N., Blanco J., Erkizia I., Fernández-Figueras M.T., Borràs F.E., Naranjo-Gómez M., Bofill M., Ruiz L., Clotet B., Martinez-Picado J. (2007). Maturation of Blood-Derived Dendritic Cells Enhances Human Immunodeficiency Virus Type 1 Capture and Transmission. J. Virol..

[87] Izquierdo-Useros N., Lorizate M., Puertas M.C., Rodriguez-Plata M.T., Zangger N., Erikson E., Pino M., Erkizia I., Glass B., Clotet B. (2012). Siglec-1 Is a Novel Dendritic Cell Receptor That Mediates HIV-1 Trans-Infection through Recognition of Viral Membrane Gangliosides. PLoS Biol..

[88] Puryear W.B., Akiyama H., Geer S.D., Ramirez N.P., Yu X., Reinhard B.M., Gummuluru S. (2013). Interferon-Inducible Mechanism of Dendritic Cell-Mediated HIV-1 Dissemination Is Dependent on Siglec-1/CD169. PLoS Pathog..

[89] Pino M., Erkizia I., Benet S., Erikson E., Fernández-Figueras M.T., Guerrero D., Dalmau J., Ouchi D., Rausell A., Ciuffi A. (2015). HIV-1 Immune Activation Induces Siglec-1 Expression and Enhances Viral Trans-Infection in Blood and Tissue Myeloid Cells. Retrovirology.

[90] Crocker P.R., Gordon S. (1989). Mouse Macrophage Hemagglutinin (Sheep Erythrocyte Receptor) with Specificity for Sialylated Glycoconjugates Characterized by a Monoclonal Antibody. J. Exp. Med..

[91] Rempel H., Calosing C., Sun B., Pulliam L. (2008). Sialoadhesin Expressed on IFN-Induced Monocytes Binds HIV-1 and Enhances Infectivity. PLoS ONE.

[92] Crocker P.R., Mucklow S., Bouckson V., McWilliam A., Willis A.C., Gordon S., Milon G., Kelm S., Bradfield P. (1994). Sialoadhesin, a Macrophage Sialic Acid Binding Receptor for Haemopoietic Cells with 17 Immunoglobulin-like Domains. EMBO J..

[93] Crocker P.R., Vinson M., Kelm S., Drickamer K. (1999). Molecular Analysis of Sialoside Binding to Sialoadhesin by NMR and Site-Directed Mutagenesis. Biochem. J..

[94] Hartnell A., Steel J., Turley H., Jones M., Jackson D.G., Crocker P.R. (2001). Characterization of Human Sialoadhesin, a Sialic Acid Binding Receptor Expressed by Resident and Inflammatory Macrophage Populations. Blood.

[95] Izquierdo-Useros N., Lorizate M., Contreras F.-X., Rodriguez-Plata M.T., Glass B., Erkizia I., Prado J.G., Casas J., Fabriàs G., Kräusslich H.-G. (2012). Sialyllactose in Viral Membrane Gangliosides Is a Novel Molecular Recognition Pattern for Mature Dendritic Cell Capture of HIV-1. PLoS Biol..

[96] Puryear W.B., Yu X., Ramirez N.P., Reinhard B.M., Gummuluru S. (2012). HIV-1 Incorporation of Host-Cell-Derived Glycosphingolipid GM3 Allows for Capture by Mature Dendritic Cells. Proc. Natl. Acad. Sci. USA.

[97] McDonald D., Wu L., Bohks S.M., KewalRamani V.N., Unutmaz D., Hope T.J. (2003). Recruitment of HIV and Its Receptors to Dendritic Cell-T Cell Junctions. Science.

[98] Yu H.J., Reuter M.A., McDonald D. (2008). HIV Traffics through a Specialized, Surface-Accessible Intracellular Compartment during Trans-Infection of T Cells by Mature Dendritic Cells. PLoS Pathog..

[99] Garcia E., Pion M., Pelchen-Matthews A., Collinson L., Arrighi J.-F.F., Blot G., Leuba F., Escola J.-M.M., Demaurex N., Marsh M. (2005). HIV-1 Trafficking to the Dendritic Cell-T-Cell Infectious Synapse Uses a Pathway of Tetraspanin Sorting to the Immunological Synapse. Traffic.

[100] Théry C., Duban L., Segura E., Véron P., Lantz O., Amigorena S. (2002). Indirect Activation of Naïve CD4+ T Cells by Dendritic Cell-Derived Exosomes. Nat. Immunol..

[101] Benet S., Gálvez C., Drobniewski F., Kontsevaya I., Arias L., Monguió-Tortajada M., Erkizia I., Urrea V., Ong R.Y., Luquin M. (2021). Dissemination of Mycobacterium Tuberculosis Is Associated to a SIGLEC1 Null Variant That Limits Antigen Exchange via Trafficking Extracellular Vesicles. J. Extracell. Vesicles.

[102] Knight S.C., Patterson S. (1997). Bone Marrow-Derived Dendritic Cells, Infection with Human Immunodeficiency Virus, and Immunopathology. Annu. Rev. Immunol..

[103] UNAIDS Data 2021. https://www.unaids.org/sites/default/files/media_asset/JC3032_AIDS_Data_book_2021_En.pdf.

[104] Spira A.I., Marx P.A., Patterson B.K., Mahoney J., Koup R.A., Wolinsky S.M., Ho D.D. (1996). Cellular Targets of Infection and Route of Viral Dissemination after an Intravaginal Inoculation of Simian Immunodeficiency Virus into Rhesus Macaques. J. Exp. Med..

[105] Miller C.J., Li Q., Abel K., Kim E.-Y., Ma Z.-M., Wietgrefe S., La Franco-Scheuch L., Compton L., Duan L., Shore M.D. (2005). Propagation and Dissemination of Infection after Vaginal Transmission of Simian Immunodeficiency Virus. J. Virol..

[106] Masurier C., Salomon B., Guettari N., Pioche C., Guigon M., Klatzmann D., Lachapelle F. (1998). Dendritic Cells Route Human Immunodeficiency Virus to Lymph Nodes after Vaginal or Intravenous Administration to Mice. J. Virol..

[107] Hu J., Gardner M.B., Miller C.J. (2000). Simian Immunodeficiency Virus Rapidly Penetrates the Cervicovaginal Mucosa after Intravaginal Inoculation and Infects Intraepithelial Dendritic Cells. J. Virol..

[108] Shen R., Kappes J.C., Smythies L.E., Richter H.E., Novak L., Smith P.D. (2014). Vaginal Myeloid Dendritic Cells Transmit Founder HIV-1. J. Virol..

[109] Trifonova R.T., Bollman B., Barteneva N.S., Lieberman J. (2018). Myeloid Cells in Intact Human Cervical Explants Capture HIV and Can Transmit It to CD4 T Cells. Front. Immunol..

[110] Hu Q., Frank I., Williams V., Santos J.J., Watts P., Griffin G.E., Moore J.P., Pope M., Shattock R.J. (2004). Blockade of Attachment and Fusion Receptors Inhibits HIV-1 Infection of Human Cervical Tissue. J. Exp. Med..

[111] Sanders R.W., de Jong E.C., Baldwin C.E., Schuitemaker J.H.N., Kapsenberg M.L., Berkhout B. (2002). Differential Transmission of Human Immunodeficiency Virus Type 1 by Distinct Subsets of Effector Dendritic Cells. J. Virol..

[112] Perez-Zsolt D., Cantero-Pérez J., Erkizia I., Benet S., Pino M., Serra-Peinado C., Hernández-Gallego A., Castellví J., Tapia G., Arnau-Saz V. (2019). Dendritic Cells from the Cervical Mucosa Capture and Transfer HIV-1 via Siglec-1. Front. Immunol..

[113] Chan R., Uchil P.D., Jin J., Shui G., Ott D.E., Mothes W., Wenk M.R. (2008). Retroviruses Human Immunodeficiency Virus and Murine Leukemia Virus Are Enriched in Phosphoinositides. J. Virol..

[114] Kalvodova L., Sampaio J.L., Cordo S., Ejsing C.S., Shevchenko A., Simons K. (2009). The Lipidomes of Vesicular Stomatitis Virus, Semliki Forest Virus, and the Host Plasma Membrane Analyzed by Quantitative Shotgun Mass Spectrometry. J. Virol..

[115] Bavari S., Bosio C.M., Wiegand E., Ruthel G., Will A.B., Geisbert T.W., Hevey M., Schmaljohn C., Schmaljohn A. (2002). Lipid Raft Microdomains: A Gateway for Compartmentalized Trafficking of Ebola and Marburg Viruses. J. Exp. Med..

[116] Feizpour A., Yu X., Akiyama H., Miller C.M., Edmans E., Gummuluru S., Reinhard B.M. (2015). Quantifying Lipid Contents in Enveloped Virus Particles with Plasmonic Nanoparticles. Small.

[117] Uchil P.D., Pi R., Haugh K.A., Ladinsky M.S., Ventura J.D., Barrett B.S., Santiago M.L., Bjorkman P.J., Kassiotis G., Sewald X. (2019). A Protective Role for the Lectin CD169/Siglec-1 against a Pathogenic Murine Retrovirus. Cell Host Microbe.

[118] Frederico B., Chao B., Lawler C., May J.S., Stevenson P.G. (2015). Subcapsular Sinus Macrophages Limit Acute Gammaherpesvirus Dissemination. J. Gen. Virol..

[119] Iannacone M., Moseman E.A., Tonti E., Bosurgi L., Junt T., Henrickson S.E., Whelan S.P., Guidotti L.G., Von Andrian U.H. (2010). Subcapsular Sinus Macrophages Prevent CNS Invasion on Peripheral Infection with a Neurotropic Virus. Nature.

[120] Ruffin N., Gea-Mallorquí E., Brouiller F., Jouve M., Silvin A., See P., Dutertre C.A., Ginhoux F., Benaroch P. (2019). Constitutive Siglec-1 Expression Confers Susceptibility to HIV-1 Infection of Human Dendritic Cell Precursors. Proc. Natl. Acad. Sci. USA.

[121] Zou Z., Chastain A., Moir S., Ford J., Trandem K., Martinelli E., Cicala C., Crocker P., Arthos J., Sun P.D. (2011). Siglecs Facilitate HIV-1 Infection of Macrophages through Adhesion with Viral Sialic Acids. PLoS ONE.

[122] Manel N., Hogstad B., Wang Y., Levy D.E., Unutmaz D., Littman D.R. (2010). A Cryptic Sensor for HIV-1 Activates Antiviral Innate Immunity in Dendritic Cells. Nature.

[123] Lahaye X., Satoh T., Gentili M., Cerboni S., Conrad C., Hurbain I., ElMarjou A., Lacabaratz C., Lelièvre J.D., Manel N. (2013). The Capsids of HIV-1 and HIV-2 Determine Immune Detection of the Viral CDNA by the Innate Sensor CGAS in Dendritic Cells. Immunity.

[124] Martinez O., Leung L.W., Basler C.F. (2012). The Role of Antigen-Presenting Cells in Filoviral Hemorrhagic Fever: Gaps in Current Knowledge. Antivir. Res..

[125] Perez-Zsolt D., Erkizia I., Pino M., García-Gallo M., Martin M.T., Benet S., Chojnacki J., Fernández-Figueras M.T., Guerrero D., Urrea V. (2019). Anti-Siglec-1 Antibodies Block Ebola Viral Uptake and Decrease Cytoplasmic Viral Entry. Nat. Microbiol..

[126] Kuroda M., Halfmann P., Kawaoka Y. (2020). HER2-Mediated Enhancement of Ebola Virus Entry. PLoS Pathog..

[127] Alvarez C.P., Lasala F., Carrillo J., Muñiz O., Corbí A.L., Delgado R. (2002). C-Type Lectins DC-SIGN and L-SIGN Mediate Cellular Entry by Ebola Virus in Cis and in Trans. J. Virol..

[128] Lasala F., Arce E., Otero J.R., Rojo J., Delgado R. (2003). Mannosyl Glycodendritic Structure Inhibits DC-SIGN-Mediated Ebola Virus Infection in Cis and in Trans. Antimicrob. Agents Chemother..

[129] Erikson E., Wratil P.R., Frank M., Ambiel I., Pahnke K., Pino M., Azadi P., Izquierdo-Useros N., Martinez-Picado J., Meier C. (2015). Mouse Siglec-1 Mediates Trans-Infection of Surface-Bound Murine Leukemia Virus in a Sialic Acid N-Acyl Side Chain-Dependent Manner. J. Biol. Chem..

[130] Sewald X., Ladinsky M.S., Uchil P.D., Beloor J., Pi R., Herrmann C., Motamedi N., Murooka T.T., Brehm M.A., Greiner D.L. (2015). Retroviruses Use CD169-Mediated Trans-Infection of Permissive Lymphocytes to Establish Infection. Science.

[131] Akiyama H., Miller C., Patel H.V., Hatch S.C., Archer J., Ramirez N.-G.P., Gummuluru S. (2014). Virus Particle Release from Glycosphingolipid-Enriched Microdomains Is Essential for Dendritic Cell-Mediated Capture and Transfer of HIV-1 and Henipavirus. J. Virol..

[132] Perez-Zsolt D., Muñoz-Basagoiti J., Rodon J., Elousa-Bayes M., Raïch-Regué D., Risco C., Sachse M., Pino M., Gumber S., Paiardini M. (2021). SARS-CoV-2 Interaction with Siglec-1 Mediates Trans-infection by Dendritic Cells. Cell Mol. Immunol..

[133] Delorey T.M., Ziegler C.G.K., Heimberg G., Normand R., Yang Y., Segerstolpe Å., Abbondanza D., Fleming S.J., Subramanian A., Montoro D.T. (2021). COVID-19 Tissue Atlases Reveal SARS-CoV-2 Pathology and Cellular Targets. Nature.

[134] Delputte P.L., Nauwynck H.J. (2004). Porcine Arterivirus Infection of Alveolar Macrophages Is Mediated by Sialic Acid on the Virus. J. Virol..

[135] Gerl M.J., Sampaio J.L., Urban S., Kalvodova L., Verbavatz J.-M.M., Binnington B., Lindemann D., Lingwood C.A., Shevchenko A., Schroeder C. (2012). Quantitative Analysis of the Lipidomes of the Influenza Virus Envelope and MDCK Cell Apical Membrane. J. Cell Biol..

[136] Blanco J., Barretina J., Ferri K.F., Jacotot E., Gutiérrez A., Armand-Ugón M., Cabrera C., Kroemer G., Clotet B., Esté J.A. (2003). Cell-Surface-Expressed HIV-1 Envelope Induces the Death of CD4 T Cells during GP41-Mediated Hemifusion-like Events. Virology.

[137] Blanco J., Barretina J., Clotet B., Este J.A. (2004). R5 HIV Gp120-Mediated Cellular Contacts Induce the Death of Single CCR5-Expressing CD4 T Cells by a Gp41-Dependent Mechanism. J. Leukoc. Biol..

[138] Denizot M., Varbanov M., Espert L., Robert-Hebmann V., Sagnier S., Elisabet Garcia E.G., Curriu M., Mamoun R., Blanco J., Biard-Piechaczyk M. (2008). HIV-1 Gp41 Fusogenic Function Triggers Autophagy in Uninfected Cells. Autophagy.

[139] Doitsh G., Galloway N.L.K., Geng X., Yang Z., Monroe K.M., Zepeda O., Hunt P.W., Hatano H., Sowinski S., Muñoz-Arias I. (2014). Cell Death by Pyroptosis Drives CD4 T-Cell Depletion in HIV-1 Infection. Nature.

[140] Monroe K.M., Yang Z., Johnson J.R., Geng X., Doitsh G., Krogan N.J., Greene W.C. (2014). IFI16 DNA Sensor Is Required for Death of Lymphoid CD4 T Cells Abortively Infected with HIV. Science.

[141] Brenchley J.M., Ruff L.E., Casazza J.P., Koup R.A., Price D.A., Douek D.C. (2006). Preferential Infection Shortens the Life Span of Human ImmunodeficiencyVirus-Specific CD4^+^ T Cells In Vivo. J. Virol..

[142] Lempp F.A., Soriaga L., Montiel-Ruiz M., Benigni F., Noack J., Park Y.-J., Bianchi S., Walls A.C., Bowen J.E., Zhou J. (2021). Lectins Enhance SARS-CoV-2 Infection and Influence Neutralizing Antibodies. Nature.

